# *Acinetobacter baumannii* producing OXA-23 detected in the Czech Republic

**DOI:** 10.1186/2193-1801-2-296

**Published:** 2013-07-02

**Authors:** Marketa Senkyrikova, Vendula Husickova, Magdalena Chroma, Pavel Sauer, Jan Bardon, Milan Kolar

**Affiliations:** Department of Microbiology, Faculty of Medicine and Dentistry, Palacky University Olomouc, Hněvotínská 5, Olomouc, 77900 Czech Republic; Institute of Molecular and Translational Medicine, Faculty of Medicine and Dentistry, Palacky University Olomouc, Hněvotínská 5, Olomouc, 77900 Czech Republic; State Veterinary Institute in Olomouc, Jakoubka ze Stříbra 1, Olomouc, 77900 Czech Republic

## Abstract

**Background:**

*Acinetobacter baumannii* is an opportunistic pathogen posing an increased risk to hospitalized persons, causing nosocomial pneumonias, urinary tract infections and postoperative infections.

**Methods:**

Between 1 December 2011 and 30 September 2012, strains of *Acinetobacter* spp. were isolated from clinical samples obtained from hospitalized patients. Susceptibility to antibiotics was determined by the standard microdilution method and phenotypic testing was used to detect the presence of serine carbapenemases and metallo-beta-lactamases. The polymerase chain reaction was used to detect the genes encoding carbapenemases. Pulsed field gel electrophoresis was used to investigate the genetic relationship among the carbapenem resistant isolates of *Acinetobacter baumannii*.

**Results:**

In three strains of *Acinetobacter baumannii* enzyme OXA-23 was detected. This positive result was confirmed by restriction analysis and sequencing. The study reported an OXA-23-producing strains of *Acinetobacter baumannii* in the Czech Republic. All three strains isolated from Military Hospital patients had a completely identical restriction profile, indicating clonal spread of a strain carrying serine carbapenemase OXA-23 in this health care facility. Moreover this was the first time the strain was detected in the country in patients who had not stayed abroad.

## Background

At present, one of the most serious issues in medicine is increasing resistance of bacterial pathogens to antimicrobial agents. This fact is associated with higher mortality and morbidity rates, prolonged hospital stays and increased treatment-related costs (Rello et al., 1994; Scaife et al., 1995; Luna et al., 1997; Micek et al., 2005; Uvizl et al., 2011; Trecarichi et al. 2011). Such negative trends have also been observed in *Acinetobacter* spp. strains. Together with isolates of *Pseudomonas aeruginosa*, *Stenotrophomonas maltophilia* and *Burkholderia cepacia* complex, these belong to the clinically most important aerobic non-fermenting Gram-negative rods. The species *Acinetobacter baumannii* is an opportunistic pathogen with increasing clinical significance, particularly in immunocompromised patients, causing nosocomial infections of the lungs, urinary tract and surgical wounds (Lee et al., 2009).

Moreover, the role of *Acinetobacter baumannii* is strengthened by relatively high resistance to numerous antibiotics which is determined by both natural and acquired mechanisms (Jeon et al., 2005). In multiresistant strains of *Acinetobacter baumannii*, the drugs of choice are carbapenems. Unfortunately, the development of resistance did not spare even this group of antimicrobial drugs, with the main mechanism being production of carbapenemases, enzymes belonging to Ambler classes B, A and D (Ambler, 1980; Bush et al., 2010). From class B carbapenemases known as metallo-beta-lactamases (MBLs), were in *Acinetobacter* spp. strains detected type of IMP, VIM and SIM enzymes (Zarrilli et al., 2009). However, resistance of *Acinetobacter baumannii* to carbapenems is more frequently caused by production of class D serine carbapenemases. These enzymes are called carbapenem-hydrolyzing class D beta-lactamases (CHDLs) (Higgins et al., 2010). The first reported acquired class D beta-lactamases with carbapenemase in *Acinetobacter baumannii* originated from Scotland and is known as OXA-23 (initially refered as ARI-1) (Scaife et al., 1995; Mugnier et al., 2010). The most common CHDL subgroups in *Acinetobacter baumannii* are OXA-23, OXA-24/40 OXA-58, OXA-143 and OXA-51 (Dijkshorn et al., 2007; Higgins et al., 2009). The gene for OXA-51 is located on a chromosome and, unlike the other OXA types able to hydrolyze carbapenems, it has a very low level of expression and does not cause resistance (Dijkshorn et al., 2007).

Nowadays CHDLs are spread worldwide and they are often involved in nosocominal infection (Peleg et al., 2008). Isolates carrying CHDLs were detected in North and South America (Peleg et al., 2008; Villegas et al.,^2007^; Merkier et al., 2008; Dalla-Costa et al., 2003; Lolans et al., 2006), Africa (Marais et al., 2004), Australia (Peleg et al., 2006), Asian area like China (Zong et al., 2008; Hsueh et al., 2002) Korea (Kim et al., 2008), Thailand (Mendes et al., 2009), Indonesia (Mendes et al., 2009) and also in European countries like the United Kigdom (Coelho et al.; 2006), the Netherlands (van den Broek et al., 2006), France (Corvec et al., 2007), Belgium (Wybo et al., 2007), Burglaria (Stoeva et al., 2008), Belgium (Bogaerts et al., 2006), Greece (Tsakris et al., 2008) or Spain (Acosta et al., 2011).

It must be stressed that resistance of *Acinetobacter* spp. to carbapenems may be caused by other mechanisms such as changes in porin expression, modification of PBPs or efflux of an antibiotic from a cell (Poirel et al., 2010).

Carbapenem resistance in *Acinetobacter baumannii* associated with OXA-type enzymes was first occurred in 2008 in the Czech republic. There were detected OXA-58-like and OXA-24-like enzymes from patients hospitalized at intensive care units (Nemec et al., 2008). Then a few years later in 2011 was in the Czech republic detected a multiresistant strain of *Acinetobacter baumannii* carrying the genes for NDM-1 and OXA-23 was detected in 2011 (Křížová et al., 2012). However, this strain was isolated in a patient who had returned from a stay in Egypt and belonged to the European (EU) clone I (Křížová et al., 2012).

This is the first report in the Czech Republic of *Acinetobacter baumannii* strains producing OXA-23 isolated from a patient who did not stay abroad.

## Material and methods

### Strain selection

Between 1 December 2011 and 30 September 2012, *Acinetobacter* spp. strains were isolated from clinical samples (endotracheal secretion, bronchoalveolar lavage, sputum, blood, urine, pus, aspirate, wound secretion, blood culture). These strains were obtained from patients hospitalized at intensive care units in the University Hospital Olomouc and Military Hospital Olomouc. The identification was performed by standard microbiology procedures including the use of the Phoenix automated system (Becton, Dickinson and Company) and MALDI-TOF Biotyper (Bruker Daltonics).

### Determining resistance to antimicrobial agents

In all *Acinetobacter* spp. isolates, susceptibility to antibiotics was determined by a standard microdilution method according to the EUCAST (European Committee on Antimicrobial Susceptibility Testing) criteria (European Committee on Antimicrobial Susceptibility Testing, 2012). The reference strains for quality controls were *Escherichia coli* ATCC 25922, *Escherichia coli* ATCC 35218 and *Pseudomonas aeruginosa* ATCC 27853. Resistance to meropenem determined by the microdilution method was confirmed by the E-test (bioMérieux, France). In case of imipenem and ertapenem, the E-test was also used to determine the minimum inhibitory concentrations (MIC).

### Phenotypic determination of carbapenemase production

**Figure 1:**
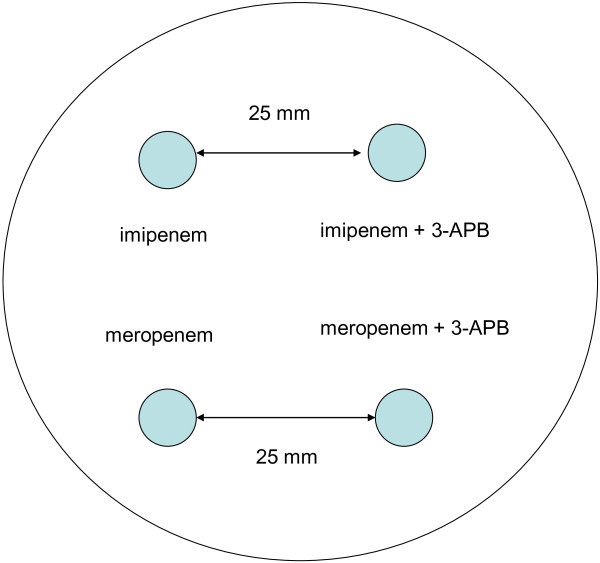
**CD test for class A carabapenemase detection.** Legend: 3-APB - 3-aminophenylboronic acid.

**Figure 2:**
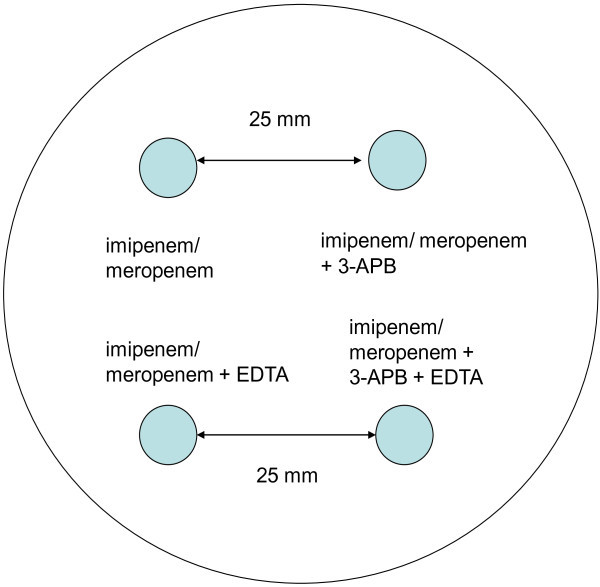
**Combined test for serine carbapenemases and metallo-beta-lactamase detection.** Legend: EDTA – ethylenediaminetetraacetic acid.

Carbapenemase production in *Acinetobacter* spp. isolates with a MIC for meropenem of >2 mg/L was phenotypically determined by the CD test (Figure 1) for detection of carbapenemases class A, combined disc (Figure 2) and modified Hodge test for serine carbapenemases and MBL detection (Lee et al., 2009; Pasteran et al., 2009; Pournaras et al., 2010).

### Genotypic determination of carbapenemase production

In *Acinetobacter baumannii* strains with a MIC for meropenem of >2 mg/L, positively phenotypically tested for carbapenemase, the relevant genes were determined. This was carried out by PCR using specific oligonucleotide primers encoding serine carbapenemases of class A (NMC, SME, IMI, KPC and GES types) and two selected class D types (OXA-23 and OXA-48). Sequences of primers for amplification are shown in Table 1. The positive control was a strain with a known content of carbapenemases (producing KPC-2 enzyme) provided by Ing. J. Hrabák, Ph.D. from the Department of Microbiology, Faculty of Medicine in Pilsen, Charles University in Prague.

**Table 1:** **Primers for detecting carbapenemases of class A and two selected class D subgroups**

Primer	Sequence	Product size (bp)	T_a_	Reference
	(5′→3′)			
SME-F	AGATAGTAAATTTTATAG	1138	50°C	Radice et al.; 2004
SME-R	CTCTAACGCTAATAG			
IMI-F	ATAGCCATCCTTGTTTAGCTC	818	50°C	Queenan et al.; 2000
IMI-R	TCTGCGATTACTTTATCCTC			
NMC1	GCATTGATATACCTTTAGCAGAGA	2158	50°C	Aubron et al.; 2005
NMC4	CGGTGATAAAATCACACTGAGCATA			
KPC-F	ATGTCACTGTATCGCCGTCT	893	60°C	Bradford et al.; 2004
KPC-R	TTTTCAGAGCCTTACTGCCC			
GES-F	GTTTTGCAATGTGCTCAACG	371	50°C	Weldhagen et al.; 2004
GES-R	TGCCATAGCAATAGGCGTAG			
OXA-23 F	AAGCATGATGAGCGCAAAG	1066	50°C	Donald et al.; 2000
OXA-23R	AAAAGGCCCATTTATCTCAAA			
OXA-48 F	TTGGTGGCATCGATTATCGG	744	50°C	Poirel et al., 2004
OXA-48R	GAGCACTTCTTTTGTGATGGC			

Legend: T_a_ – annealing temperature.

Strains selected for gene detection were inoculated onto Mueller Hinton agar (Trios, Czech Republic) and aerobically cultured at 37°C for 18 hours. 1–2 colonies from this fresh culture were resuspended in 100 μL of sterile water and heated at 95°C for 10 minutes. This was followed by centrifugation at 13,000×*g* for 2 minutes. The obtained supernatant served as a template for subsequent PCR using the Robocycler Gradient 96 Temperature Cycler with specific primers shown in Table 1.

The obtained amplicons were subsequently separated on 1.5% agarose gel and compared with a DNA molecular weight marker (Top-Bio, Czech Republic). Then, OXA-23-positive PCR products were cleaved with the *Hph*I restriction endonuclease (New England Biolabs, Great Britain) and separated on 1.5% agarose gel, with cleaved fragment sizes being compared with the molecular weight marker. The amplified gene sequence was confirmed by direct sequencing of a PCR amplicon provided by Elisabeth Pharmacon (Czech Republic) and by comparing the obtained DNA sequence in BLAST (Basic Local Alignment Searching Tool, National Center for Biotechnology Information, http://www.ncbi.nlm.nih.gov).

### Determination of epidemiological relationship

To determine epidemiological relationship of strains with positive genotypic assay results, macrorestriction profiles of genome DNA were compared by pulsed-field gel electrophoresis (PFGE).

The PFGE analysis was carried out with bacterial DNA isolated from culture freshly grown on Mueller Hinton broth as described previously (Husičková et al., 2012). Bacterial DNA was then cleaved with the *Sma*I restriction endonuclease (Takara, Japan). The restriction fragments were separated in 1.2% agarose gel using the CHEF-DRII (Bio-Rad, USA) under the following conditions: 5 V/cm, switch interval 2–20 seconds, for 20 hours at 14°C in 0.5 × TBE buffer. The gel was stained with ethidium bromide at 0.75 μg/ml for 1 h and visualized by UV transllumination. A 50–1,000 kb Pulse marker (Sigma, USA) was used to determine the size of DNA fragments. The PFGE results were analysed with GelCompar II (Applied Maths) software.

## Results

**Figure 3:**
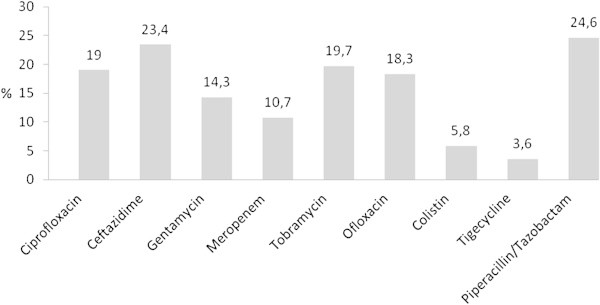
**Resistance of*Acinetobacter*spp. to selected antimicrobial agents (percentages).**

Over the study period, a total of 166 *Acinetobacter* spp. strains were isolated in the two participating hospitals. Figure 3 shows the resistance to antimicrobial agents in a group of 140 isolates assumed to play a role in the etiology of the particular infection. The results revealed a prevalence of meropenem-resistant strains of 10.7%.

From the group of 140 strains, a total of 15 strains were found to be resistant to meropenem using the standard microdilution method. The phenotypic assay demonstrating production of carbapenemases (modified Hodge test) was positive in 3 strains. In these 3 strains, MICs to selected antimicrobial agents (ertapenem, imipenem and meropenem) were determined by the E-test, as shown in Table 2.

**Table 2:** **MICs (in mg/L) of the tested carbapenems**

Strain no.	MIC(mg/L)		
	Meropenem	Imipenem	Ertapenem
**21678/C**	12	16	>32
**12257/C**	>32	16	>32
**15848/C**	>32	>32	>32

E-test MICs testing.

All the three *Acinetobacter baumannii* strains originated from the Military Hospital. Strain no. 21678/C was isolated from blood culture of a patient with bloodstream infection staying at a department of anesthesiology and intensive care medicine. Samples nos. 12257/C and 15848/C were isolated from endotracheal secretions collected from two patients with late-onset ventilator-associated pneumonia hospitalized at a department of chronic intensive care.

Genetic determination of the presence of resistance genes confirmed the presence of a gene encoding class D carbapenemase, namely OXA-23 enzyme in all three strains. Restriction fragment length polymorphism analysis in positive PCR amplicons and final confirmation by direct sequencing showed the presence of OXA-23 enzyme in all three *Acinetobacter baumannii* strains. Comparison of macrorestriction profiles of genomic DNA using PFGE revealed that the strains had an identical restriction profile. This suggested clonal spread of a genetically identical strain.

## Discussion

Class D serine carbapenemase in an *Acinetobacter baumannii* strain was first reported in Scotland in 1985, that is, before imipenem started to be widely used in clinical practice (Paton et al., 1993; Carvalho et al., 2011). This enzyme, originally called ARI-I, was sequenced and subsequently labeled as OXA-23 (Scaife et al., 1995; Mugnier et al., 2010).

Until now, this enzyme has been reported in *Acinetobacter baumannii* strains throughout the world (Mugnier et al., 2010).

In the Czech republic a multiresistant strain of *Acinetobacter baumannii* carrying the genes for NDM-1 and OXA-23 was detected in 2011. However, this strain was isolated in a patient who had returned from a stay in Egypt and belonged to the European (EU) clone I (Křížová et al., 2012). Thus, the above description of *Acinetobacter baumannii* strains producing OXA-23 is their first report in the Czech Republic in patients who did not stay abroad, suggesting their domestic origin. All three strains isolated from Military Hospital patients had a completely identical restriction profile, indicating clonal spread of a strain carrying serine carbapenemase OXA-23 in this health care facility.

Increasing resistance of bacterial pathogens to antimicrobial agents poses a severe threat to management of many infections. Of particular risk is the rise in the use of carbapenems resulting from a high prevalence of ESBL- and AmpC-positive Enterobacteriaceae which may determine the development of resistance to this group of still effective drugs (Coelho et al., 2004). Our data suggest that in the case of *Acinetobacter* spp. strains, the prevalence of carbapenem-resistant isolates is low. However, this situation needs to be carefully monitored and if this type of resistance spreads it must be adequately analyzed using modern molecular biology methods.

### Consent

Written informed consent was obtained from the patient for the publication of this report and any accompanying images.
